# A Spiking Neural Network Builder for Systematic Data-to-Model Workflow

**DOI:** 10.3389/fninf.2022.855765

**Published:** 2022-07-13

**Authors:** Carlos Enrique Gutierrez, Henrik Skibbe, Hugo Musset, Kenji Doya

**Affiliations:** ^1^Neural Computation Unit, Okinawa Institute of Science and Technology Graduate University, Okinawa, Japan; ^2^Brain Image Analysis Unit, RIKEN Center for Brain Science, Wako, Japan

**Keywords:** spiking neural networks, computational brain modeling, neural simulation, web application, data-to-model workflow, collective intelligence

## Abstract

In building biological neural network models, it is crucial to efficiently convert diverse anatomical and physiological data into parameters of neurons and synapses and to systematically estimate unknown parameters in reference to experimental observations. Web-based tools for systematic model building can improve the transparency and reproducibility of computational models and can facilitate collaborative model building, validation, and evolution. Here, we present a framework to support collaborative data-driven development of spiking neural network (SNN) models based on the Entity-Relationship (ER) data description commonly used in large-scale business software development. We organize all data attributes, including species, brain regions, neuron types, projections, neuron models, and references as tables and relations within a database management system (DBMS) and provide GUI interfaces for data registration and visualization. This allows a robust “business-oriented” data representation that supports collaborative model building and traceability of source information for every detail of a model. We tested this data-to-model framework in cortical and striatal network models by successfully combining data from papers with existing neuron and synapse models and by generating NEST simulation codes for various network sizes. Our framework also helps to check data integrity and consistency and data comparisons across species. The framework enables the modeling of any region of the brain and is being deployed to support the integration of anatomical and physiological datasets from the brain/MINDS project for systematic SNN modeling of the marmoset brain.

## 1. Introduction

Large amounts of diverse brain data are being generated from multiple brain science projects around the world (Markram et al., 2011; Okano et al., 2016; Abbott, 2021). However, to understand the functions of the brain, it is necessary to integrate such diverse experimental data as neural network models and to analyze dynamics and information transfer through systematic simulations. As an approach to effectively utilize experimental data, projects are promoting development of tools for brain modeling, such as the virtual brain (Sanz Leon et al., 2013), NetPyNE (Dura-Bernal et al., 2019), the Brain modeling toolKit (Dai et al., 2020), NEST Desktop (Spreizer et al., 2021), or PhysioDesigner (Asai et al., 2012). Besides that, a range of tools has been proposed as well for facilitating model description and supporting workflow-related processes, such as NeuroML (Gleeson et al., 2010), Mozaik (http://neuralensemble.org/docs/mozaik), SNNtoolbox (Rueckauer et al., 2017), Nengo (Bekolay et al., 2014), pypet (Meyer and Obermayer, 2016), NeuroManager (Stockton and Santamaria, 2015), and others.

**Figure 1:**
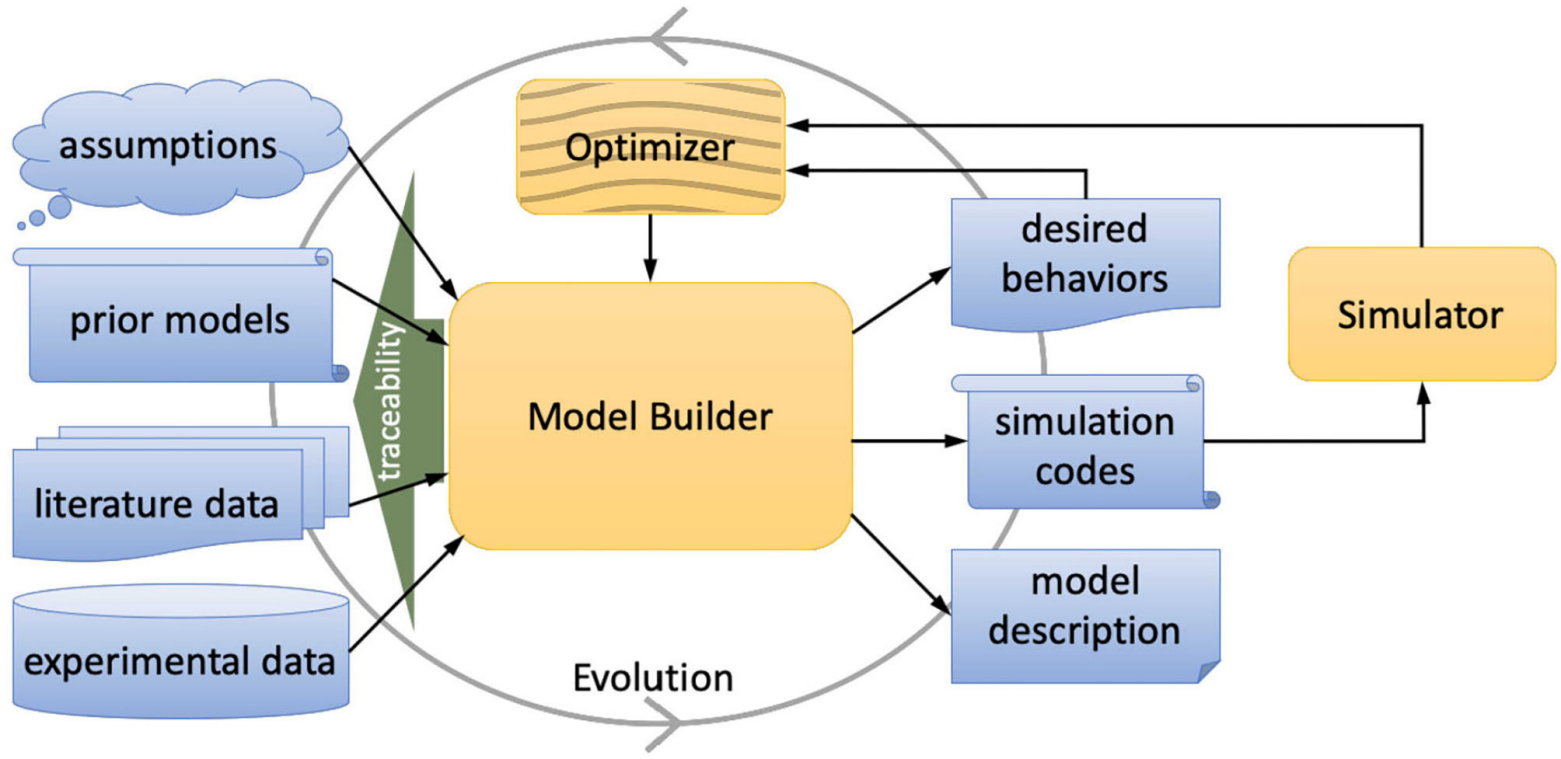
SNNbuilder conceptualization. Different data modalities are loaded through web GUI. The model builder manages the data-to-model process, generating files for systematic simulation (optimization is not included in the current release). This agile process allows the evolution of models.

In building realistic brain models, it is necessary to systematically incorporate experimental data, published data in the literature, parameters from prior models, and theoretical or mechanistic assumptions (Figure 1). Because most models have uncertain parameters, tuning them by comparing simulated model behaviors and experimental observations and/or functional assumptions is also an essential process in modeling. Performing such model building and systematic verification by maintaining traceability of the bases for parameter settings is essential for accountability, reproducibility, and future revision (evolvability) of the model.

SNNbuilder (https://snnbuilder.riken.jp) is a web-based collaborative tool for data-driven modeling by spiking neural networks (SNN). It allows the collection and management of model parameters of any region of the brain for any species, by virtue of its generic data representation using tables, attributes, and relations in a common database.

SNNbuilder uses neuron and synapse models following the state-of-the-art neural network simulator NEST (Hahne et al., 2021) and manages the data-to-model passage using a set of transfer functions to generate neural parameters and connection rules. Partial data are completed automatically with default values, while alternative and multiple data items from different sources and users are combined as a collective estimation. Model parameters can be specified as “fixed” or “to-optimize” values, as well as assumptions or prior values. Every value is linked to references for traceability.

SNNbuilder creates a model description as a JSON (JavaScript Object Notation) file with full specifications and data modalities, including desired behaviors labeled as “objectives.” The framework also generates simulation code in PyNEST (the python bindings of the NEST simulator) for building and simulating SNN models.

SNNbuilder allows an agile modeling workflow, with a primary focus on model specifications. Starting with the main parameters, a model can be created, systematically tested, and can gradually evolve with further data and collaborative contributions. The framework is designed as a web-based, multi-user application with an intuitive graphical user interface (GUI). Considering other tools, as far as we know, SNNbuilder constitutes the first attempt in offering a shared place where many users get together for building collaboratively common models. This is a straightforward way to organize users toward one of the most challenging and important tasks: modeling the complex network of the brain in a thoroughly sustainable manner.

Japan's Brain/MINDS project (Brain Mapping by Integrated Neurotechnologies for Disease Studies, https://brainminds.jp/en/; Okano et al., 2016) is building a multi-scale marmoset brain map with structural and functional imaging. Images are obtained from diffusion MRI, systematic tracer injections (Skibbe et al., 2019; Gutierrez et al., 2020; Watakabe et al., 2021), and many types of fluorescent calcium imaging. SNNbuilder seeks to integrate such diverse, large-scale data into computational modeling, and open data and tools from other brain projects.

## 2. Design

SNNbuilder is designed as a “web-app,” developed using .NET and C#, an open-source developer platform. Its database runs on MySQL, an open-source relational database management system (DBMS). The selection of a web environment for brain modeling, responds to the importance of the internet as a common shared space that enables users to access from remote locations, perform modeling tasks transparently, and share up-to-date models. For straightforward online collaboration, a login system manages accesses and permissions (see section 3.10).

### 2.1. Design Principles

From its conceptualization, the framework takes into account modeling principles, as follows:

**Fairness and transparency:** our framework allows linking model parameters with experimental data, database entries, or scientific publications. References as DOIs (digital object identifier) or URLs can be recorded for every detail of a model. Model descriptions and simulation codes are open to the research community through the web app.

**Reproducibility:** the framework provides automatic generation of simulation code. Models can be re-built with different choices of source data, and results can be reproduced by simulation of generated codes.

**Sustainability:** upon the emergence of new papers or experimental data, SNNbuilder allows model updates, such as parameter additions, modifications, and deletions. Rather than building a model for just one point in time, our framework facilitates sustained model evolution.

**Collective action**: similar experimental studies may produce dissimilar data in different laboratories and at different times. Our framework allows the loading of several values for the same data attribute. In such a way, better parameter settings may be selected by collective contributions from various modelers.

### 2.2. From Brain Biology to Database Structure

Depending on the region of the brain, degrees of detail and scale, SNN models can incorporate various types of neurons and synapses, as thousands, millions, or billions of components. For that reason, we designed a generic database to support a diversity of models, species, scales, and growing data.

To set up a comprehensive database structure for any model of the brain, we first identified, from brain biology, the most important generic objects that “produce” data, similar to specifying the main components and features on software development projects. We described those “data provider” objects and their relations as entities with multiple data attributes and connections using Entity-Relationship (ER) modeling (Chen, 1976, 2002). ER modeling is commonly used in software engineering for the representation of business needs and processes and provides a business-recognized framework to define the information structure of a relational database.

**Figure 2:**
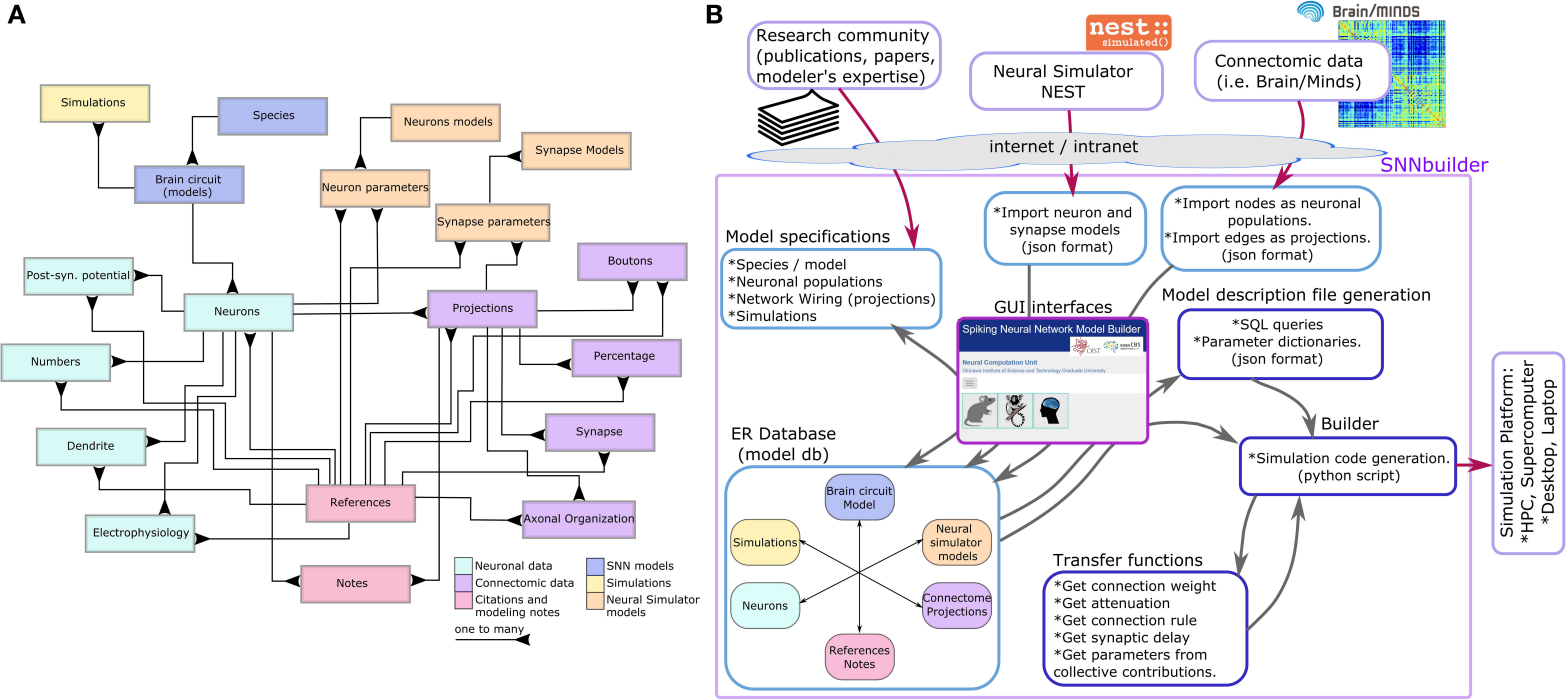
The generic ER (entities and relations) data model **(A)** is able to capture specifications of any region of the brain for any species. Closely related entities are shown as linked groups (color code). The modeling workflow **(B)** runs on GUI. Inputs correspond to research publications, modeler expertise, connectomic data, and models from NEST simulator. Specifications are stored in the database. A builder function performs data mapping as NEST parameters using either transfer functions or direct assignment, creates a model description file, and generates code for simulation.

From our analysis, six main entity groups were identified (Figure 2A):

- SNN models: the description of a brain circuit or region to be modeled for a certain species.
- Neuronal data: neuron types or neural populations, including relevant anatomical, morphological, and physiological characteristics.
- Connectomic data: projections between neural populations, along with anatomical and morphological details of network wiring.
- Citations and modeling notes: for reporting origins of data (references), such as DOIs or other URLs, and recording memos over the modeling workflow.
- Neural simulator models: a generic structure to manage data attributes of neuron and synapse models of a neural simulator, like NEST.
- Simulations: for specification of multiple simulations, including stimuli and recordables.

Entities and relations were created in MySQL as tables with primary and foreign keys to preserve data integrity and consistency. The database design applies to any other relational DBMS as well.

## 3. Model Building Workflow

**Figure 3:**
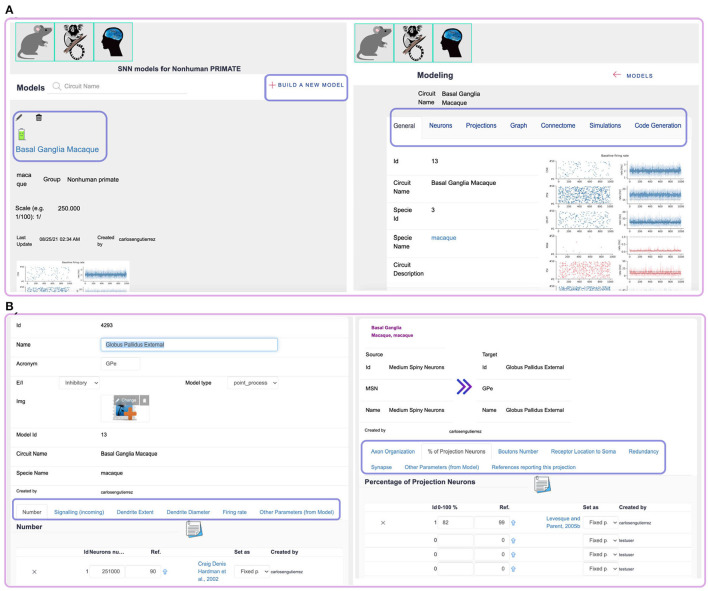
SNNbuilder GUI. **(A)** Left: front-end for species selection and model creation. Right: the modeling workflow is organized by option tabs. **(B)** Left: data requirements for neurons include numbers, PSP, dendrite characteristics, firing rates, and NEST neuron parameters. Right: projection specifications incorporate axonal properties, bouton counts, receptor locations, redundancy, synapses, and NEST synapse parameters.

The modeling workflow runs on the GUI (Figure 2B), allowing database updates in real time. The process begins by selecting a species, creating a new model instance by the option “Build a new model” and adding a description of the targeted neural circuit or brain region (Figure 3A left). At this initial step, the system generates a “model id” for identifying uniquely the model.

Model scale, in relation to biological size, is also specified. Whereas, modelers indicate realistic anatomical data, such as numbers of neurons, bouton counts, axonal domains, a scale parameter adjusts all numbers at code generation time. The scale is relevant for implementation purposes; however, limitations on the reducibility of network sizes (Van Albada et al., 2015) indicate the importance of realistic numbers of neurons and synapses. Given the available computational resources, small scales may run on laptops or desktop computers, while large scales run on servers.

After the initial settings, model specifications (input data) are required (Figure 3A right): details of neural populations, projections or connectivity data, and models from NEST simulator.

### 3.1. Neural Populations

A neural population is created by an insert operation. This records the population name (or cell type), its excitatory or inhibitory regime, and if available, a related image. Further data requirements are arranged in a tabbed document interface (Figure 3B left), organized as Liénard and Girard (2014), as below:

- Number: the number of neurons *N* within a nucleus at a real scale, considering a single brain hemisphere.
- Signaling: this refers to the neurotransmitter receptor type (AMPA and NMDA for excitatory/glutamate neurotransmitter, GABA for inhibitory/gaba neurotransmitter) of the neuron. Likewise, the PSP (post-synaptic potential) amplitude or change *V*_*n*_ (*mV*) caused by a single spike mediated by a neurotransmitter *n* to the membrane potential at the location of the receptor (synapse), and its rise time *t*_*V*_*n*__ (*ms*).
- Dendrite extent: the average maximal extent *l* (μ*m*) of the neuronal dendritic field.
- Dendrite diameter: the mean diameter *d* (μ*m*) of neuronal dendrites along their entire lengths.
- Firing rate: a biologically plausible range $\left[\phi_{0}^{s},\phi_{1}^{s}\right]$ of the neural population mean firing rate (*Hz*) for different states *s*: resting state, excitation (or functional) state, maximum activity, and disease condition. Firing rate is considered a cost function (or objective) and labeled accordingly (see data flags). The future work will consider the integration of an optimization process (see Current limitations).
- Other parameters: parameters of a selected NEST neuron model. Every neural population is paired to a NEST model by an “import from model” operation that selects the neuron model and recalls NEST parameters with default values. After the import, parameter values can be updated. See section 3.3 for more details.
- Objectives/Metrics: it corresponds to user defined objectives and metrics. A configurable set of objectives is available in the main menu (**Figure 7**.3), including, for example, coefficient of variation, inter-spike interval, fano factor (Rajdl et al., 2020), and other arbitrary targets. In this tab section, multiple objectives can be selected and their target values or metrics specified, including the related references. Objectives/metrics are later generated as comments on the simulation script (see Current limitations).

### 3.2. Projections

An insert operation facilitates data-entry for model connectivity. Projections link the source and target neural populations created in the previous step. Their connectivity is defined by connection rules specified per source-target pair. Further data requirements are organized in a tabbed document interface as well (Figure 3B right), with a structure similar to Liénard and Girard (2014):

- Connection rule: it defines the connectivity modality based on NEST connection rules for spatially-structured networks. Indegree- and outdegree-based rules are made available and probability-based, such as constant probability and distance-depended Gaussian probability rules. Transfer functions (see Appendix A) define the indegree and outdegree parameters, whereas a constant probability or SD parameters can be specified by GUI in the case of probability-based rules.
- Axon organization: source-target connection type can be focused or diffused, so synapses are taken from (or made to) neurons within narrow or wide spatial domains, respectively (i.e., a circular or spherical mask). The domain refers to the mean radius (*mm* or in units relative to the spatial organization of neurons) of a circle (sphere) approximating the 2D shape (3D-volume) of axonal arbors.
- Percentage of projection neurons: the proportion of neurons *P*∈[0, 100]% in the source population with axons connecting the target population.
- Bouton number: the mean number of axonal varicosities (or boutons) α where synapses may occur. A biologically plausible range is defined for the sake of exploration; thus, bouton counts are considered as “to-optimize” parameters (see Data flags and Current limitations).
- Receptor location to soma: the mean distance *r* to the soma of synaptic receptors along dendrites, expressed as a proportion of dendrite extent *l*. It takes values within ranges for exploration: proximal *r*∈[0, 0.2), medial *r*∈[0.2, 0.6), and distal *r*∈[0.6, 1]. It is considered a “to-optimize” parameter (see Current limitations). This parameter is used to calculate an attenuation of the connection weights (see Transfer functions). Specifying *r* as “None” removes attenuation from connection weights.
- Redundancy (Girard et al., 2020): the mean number ρ of contacts made by axons on each dendritic tree. It is a number between [1, ν], with ν being the total number of synapses converging on a single neuron. Redundancy can be used to adjust the number of connections and their strength, especially for scaled-down model simulations (see Appendix A: Transfer functions).
- Synapse: records the communication delay (*ms*) (or axonal delay) of a projection and the corresponding connection weight. If defined, synapse data overwrite the default values of the selected NEST synapse model (see Appendix A: Connection weight).
- Other parameters: parameters of a selected NEST synapse model. Similar to the case for neural populations, every projection is paired to a NEST model by an “import from model” operation that transfers parameters with default values to the projection for further update. See the section “Models of a neural simulator” for more details.

### 3.3. Models of a Neural Simulator

SNNbuilder uses neuron and synapse models following those of NEST (Hahne et al., 2021), a state-of-the-art simulator for SNN models that focuses on accurate dynamics, varieties of network structure, and scalability for large-scale simulation. NEST provides more than 50 neuron models, over 10 synapse models, and an active support and global community.

**Figure 4:**
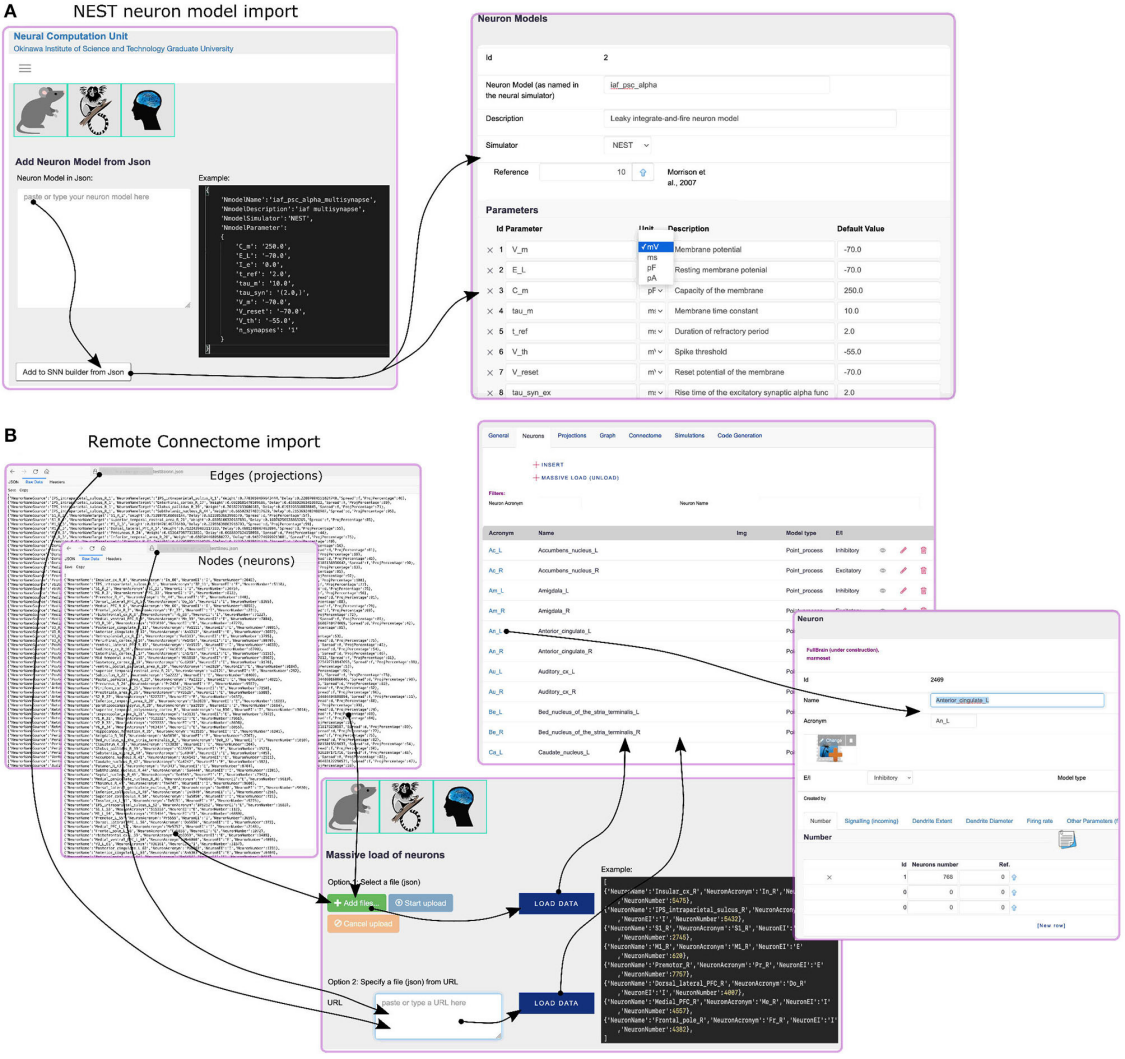
Data load. **(A)** Left: NEST models are incorporated using JSON format data entry in the edit-box area. The required format is shown in the dark background area. Right: after data loading, parameter default values can be updated. **(B)** Import function for connectomic data. Left: connectome nodes and edges are mapped as neuronal populations and projections, respectively; thus, input data are prepared as two JSON files. Center: options for data loading include a file upload or import-from-URL functions. The dark background area shows an example of the required format. Right: after loading to the database, neurons and projections can be explored and updated.

Parameters from NEST neuron or synapse models can be added using the insert or import functions. The latter reads a JSON formatted NEST model from a web edit-box and imports parameter names, descriptions, and default values to the database. The GUI allows manual data-entry or “cut and paste” commands (Figure 4A left). The data structure for neuron and synapse models is generic at the database level (Figure 4A right), so it can support several neural simulators.

### 3.4. Data Sources for Modeling

At the time of this report, paper surveys, identification, and manual loading of parameter values are the main activities for model specification at SNNbuilder. Nevertheless, its “online” condition supports potential integration with resources available over the internet, for example, knowledge graphs, public databases, or web services that provide data on-demand, for example, connectomic data (see section 5 and Supplementary Figure B.1). Connectomes are frequently generated as open sources for the advancement of science. Tracer studies, DTI (diffusion tensor image)-based fiber tracking, and functional MRI (magnetic resonance image) data are frequently arranged as region-level (mesoscale data) connectome matrices, where nodes correspond to brain regions and edges to their connections.

To allow such data integration from external sources, our system is prepared to import connectomic data from remote URLs or file-upload. The connectomic data must be provided in JSON format and separated into two files (Figure 4B left) for mapping: (i) nodes as neural populations, with specifications (if available) such as population name, number, excitatory/inhibitory regime, and others; and (ii) edges as projections, including available specifications for the axonal delay, connection weight, and others. These functions are available in the GUI (Figure 4B center). They provide a straightforward way for incorporating connectomic data and rapid model creation. Moreover, after importing, the user can add additional specifications, implement modifications, assign NEST neuron models, and other improvements (Figure 4B right). It is also possible to integrate different data scales (micro, meso, and macro). Data import reduces manual work considerably.

### 3.5. Data Flags

Flags label characteristics of the data. By default, high-confidence and frequently reported data are considered “fixed parameters,” such as neuron numbers, dendrite extent, and diameter, post-synaptic potentials, etc. Parameters, such as axonal bouton counts, and average location of synapses along dendritic trees are considered “to-optimize parameters” and defined as ranges of values for exploration. In the case of multiple entries for the same parameter, a “deactivated” flag is available to “remove” outliers and low-confidence values (**Figure 8**.11). Several activated parameters are possible, a collective “contribution” is calculated in such a case. For multiple numerical values, the average is used (Supplementary Figure B.2); in the case of non-numerical multiple values (categories), the appearance frequency is computed as an important index, with the highest frequency value as the collective outcome.

Electro-physiological constraints, such as mean firing rates, are defined as intervals of plausible neural activity, and labeled as “objective functions,” crucial for comparisons with simulated neural activity (see Current limitations).

Flag assignment depends on modeler criteria. It is recommended to distinguish between well-known data and poorly documented or inconsistent data from different sources.

### 3.6. References and Notes

Paper survey-based data entry allows recording of relevant parameter values and data providers and references for traceability. SNNbuilder enables acknowledgment of every detail attached to a model using DOIs or other URLs. Source publications can be accessed and examined directly from the framework GUI (Supplementary Figure B.2).

In addition, the GUI includes edit-boxes for digital notes, memos, or comments at every web tab of the workflow. Thus, free-text recording into the database facilitates the creation of a “diary” or “logs,” a common practice among researchers.

### 3.7. Network Viewers

**Figure 5:**
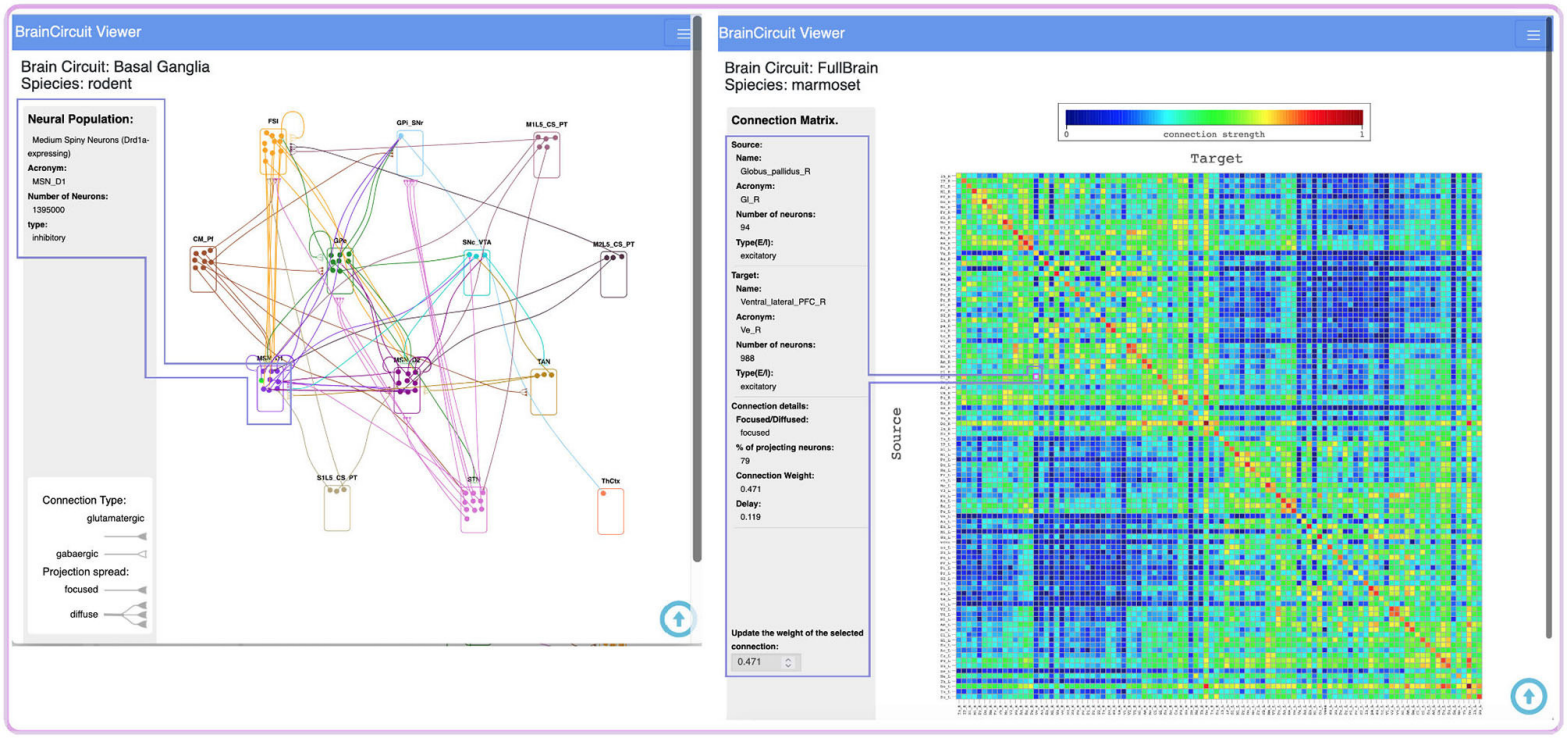
Network viewers. Graph (left) and matrix (right) viewers for exploration of model specifications with interactive data retrieval (area enclosed by blue lines). The latter enables selecting a connection within the matrix and upgrading its weight.

The GUI enables listing and navigating through model specifications, such as neurons and projections; however, to explore a model as a whole, viewers are also helpful (Figure 5). A graph-viewer visualizes neuronal populations as boxes (nodes), and projections as edges linking the boxes. The interactive nature of the viewer enables graph exploration and content retrieval from the database.

An additional viewer implements a 2D-matrix visualization for the exploration of connectivity data, such as source and target populations, axonal delays, spatial connection domains, and other network-wiring details. This interface allows straightforward modification of connection weights.

### 3.8. Simulation Settings

Model simulation criteria are specified by GUI. The main specifications include a description of the simulation, the time resolution (ms), the simulation time (ms), and the number of computational threads. In addition, a common spatial domain for neuron positioning is defined for the sake of consistency and robust simulations in NEST. This version of SNNbuilder supports spatial boundaries (minimum and maximum coordinate values) for randomly and uniformly organized neuron positions in 2D or 3D. Since multiple simulations can be specified for a certain model, different spatial arrangements can be tested.

Specifications of connection weight values might result weak in relation to other parameter values, like membrane resistances, for driving network dynamics during simulation; or too strong, leading to extreme network activity. In those cases, a multiplicative factor affecting the absolute value of all synaptic weights can be defined by the user, called synaptic scaling gain. In this way, simulations can be performed while maintaining, relatively, the specified connection strengths of the neural network model.

Besides simulation settings, details of the stimuli and recordable are also defined. Several simulations can be specified; however, only one should be activated by assigning the corresponding data flag for code generation. Otherwise, the first active simulation is considered at the PyNEST script.

#### 3.8.1. Stimuli

Stimuli are designed as independent spike trains from NEST Poisson generators (see Current limitations) and specified by GUI (**Figure 9**.26). A Poisson generator is created per target population with configurable firing rate (Hz), connection weight, axonal delay (ms), and the start and stop times (ms) of the stimulation, along with its scope. The scope refers to either Poisson spikes trains are sent to all neurons in the target (global scope) or to a spatially-bounded subset of neurons. The spatial bounds are defined by a point (position coordinates) and a radius parameter, which determines the neurons within a circle or sphere under the stimuli.

#### 3.8.2. Recordables

There exist two NEST-based recordable options for spikes and membrane potential that define what gets recorded during simulation time. Multiple recordable can be specified, with a single one targeting a single neural population. Spike-type recordable stores the spike times of all neurons at the target population; while membrane potential-type recordable selects, at random, a single neuron for recording the evolution of its membrane potential. Recordables generate, automatically, output data files.

### 3.9. Model Description and Code Generation

The workflow's final step corresponds to procedures for organizing SNNbuilder output. This includes the generation of a comprehensive list of model specifications, parameter passage to NEST models, and the generation of simulation code for creating neural populations, recording devices, network wiring, and stimuli.

Automatic generation of code is practical for immediate testing, different model configurations, and versions. High-level programming skills are not required and modeling time is used mainly for definition of biological constraints.

Model description and simulation code files are made available through 3 sequential processes (**Figure 9**.27) implemented in Flask (Grinberg, 2018), a python-based web development framework, and executable on GUI:

(1) Get parameters: for a particular model, this process runs SQL (structured query language) queries on the database and gathers the previously specified data for that model. Retrieved data are converted to python dictionaries and arranged in a single JSON file as the model description. In this step, queries make use of the data-flag specifications to filter parameters and compute collective contributions. Parameter values labeled as “deactivated” are not considered. In the case of multiple numerical values loaded for a specific parameter, the average value is considered as the collective outcome (Supplementary Figure B.2) and computed at query time. In the case of multiple categorical data, category frequencies are calculated as an “importance index.” The most weighted index is selected for parameter initialization. Queries may retrieve dictionaries with “None” records for parameters with no available data. In such cases, default settings are assigned in the next step.

(2) Code build: a builder function takes the JSON file generated at 1) applies transfer functions (see Appendix A) and the specified scale and creates the simulation script in PyNEST for NEST 3. For robust simulations, the builder generates straightforward lines of code (LOC) in the following sequence:

- Initialization: includes LOC for importing the necessary python packages. NEST kernel initialization, and the definition of global variables.
- Creation of neural populations: the process takes parameter values and creates LOC for initialization of neural populations. Parameters from (1) are mapped to NEST neuron and synapse models, updating default values. NEST defaults remain when “None's” are present at parameter specifications. Neuron numbers are adjusted based on the defined scale parameter. Signaling and PSP values set up neuron receptors and synaptic delays. Neuron positions are created in 2D or 3D space, by using a uniform random distribution, within spatial bounds defined at the simulation settings. Given the specified NEST neuron model and its parameters, LOC for the creation of the neural population is generated.
- Network building: this takes network-wiring details from specifications at (1). For connected population pairs, parameters are mapped to NEST synapse models and connection dictionaries, and LOCs are created for connection rules. For more details on connection weight definition and connection rule parameters, see Transfer functions (Appendix A).
- Stimuli, recordables, and simulation: given the specified stimuli and targets, the process generates LOCs for the creation of Poisson spike-train generators and initialization of their firing rates, spatial scope, and other parameters. Additional LOC for the creation of recording devices of neuronal activity (spikes) or membrane potentials are also included. Finally, the process creates LOC for NEST simulation commands with a defined biological time.

The tool does not include on-line code execution or execution management on its first release (see Current limitations and Discussion).

(3) File download: this takes the results from 1 (model description as JSON file) and 2 (simulation code as python script file), packs them into a zip file, and delivers them. Although the python script runs stand-alone, the specification file is made available for future parameter optimization (see Current limitations).

### 3.10. Collective Intelligence

The online and centralized database aspects of our approach allow a modern form of collaboration called “collective intelligence.” SNNbuilder is designed for multi-user access. Another important feature is the assignment of multiple values for the same parameter. Diverse input values for a single parameter improve its reliability (Supplementary Figure B.2), see Data flags and Model description and code generation sections). Over time, settings evolve to better values through different contributions of more users and new data, gradually converging to the most realistic ones.

These characteristics promote “collective intelligence,” where humans (and computers) working together act much more intelligently in a collective way than individually (Malone, 2018). As demonstrated by crowd-sourcing experiences (Brabham, 2013), a “bigger brain” works better than a small one. By this collaboration scheme, better models of the brain can be collectively built and shared online across the scientific community. In addition, SNNbuilder includes functions for maintaining digital notes or memos (see Reference and notes section), available at every tab of the GUI (Supplementary Figure B.2). In this way, users can record and share comments, questions, and logs over the workflow.

To support this scheme, the application implements a login system for user identification and automatic labeling (tags) of user contributions. When a model is created (Figures 3A, **7**.3), the owner has the choice to “open” the model to the community; in that case, multiple users can visualize, add or update records, and generate simulation code. Otherwise, the model remains close, and only the owner can access it to perform updates. Every record is owned by a specific user. Security rules disable the deletion of different user contributions; thus, only self-owned records can be removed or disabled.

### 3.11. Current Limitations

The present release of our work reports some limitations not yet solved or implemented.

Specifications are mainly fixed parameter values (numerical, categorical, or descriptions). Detailed models may require distribution-based values for some parameters, such as connection weights, synapse locations, resting membrane potentials, which are not yet included (see section 5). Complex experimental settings or detailed models may require the incorporation of functional-based metadata for setting up neuronal parameters and connectivity features; however, our application does not support that aspect.

The firing rate specifications can take numerical values and description tags indicating the “state” related to the neural activity, for example, resting, excitation, and disease states; however, the state is not linked to a certain stimulation protocol for its effective simulation. In the present release, states are enabled only for the characterization of the targeted activity.

Specifications of a model cannot be re-used by other models. SNNbuilder considers constantly evolving models; thus, parameter history is not maintained and the latest specifications are taken at code-generation time. Model versioning is not implemented (see Discussion). Data modifications, additions and deletions from multiple users are not tracked over the building workflow; however, ownership records are maintained for acknowledgment of the different contributions.

The current version of our tool provides a subset of the available NEST connection rules. Stimuli are defined using Poisson spike trains, there is no other stimulus modality implemented. Optimization is also not yet included; nevertheless, the database structure was designed to support an optimization engine (see Discussion). The system allows the import of connectomic data; however, the data require preparation in a specific JSON format (see section 5). Furthermore, there is no functionality for accessing HPC resources; therefore, simulation code cannot be executed within SNNbuilder. Code execution steps are managed by the user.

## 4. Modeling Examples

**Figure 6:**
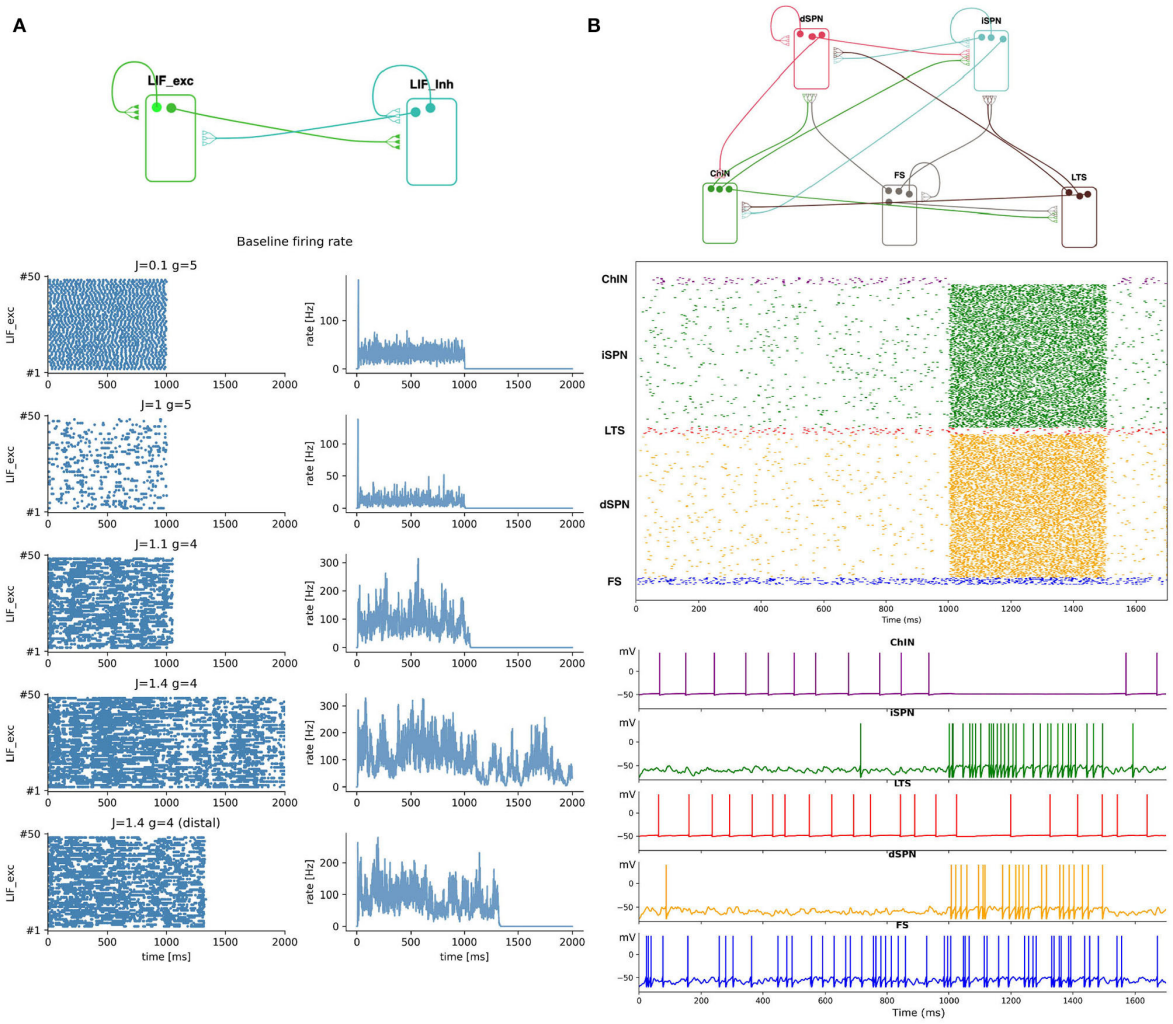
Example models. **(A)** Cortical network simulations with different values of the coupling strength between excitatory and inhibitory populations. Strong coupling allows self-sustained asynchronous irregular activity (SSAI) after stimulus offset (1,000 ms). A single change of the receptor location (synapse) to soma, from proximal to distal (plot at bottom), reduces SSAI lifetime. **(B)** Striatal microcircuitry. Rasters correspond to baseline activity of the striatal nuclei (0–1,000 ms), as well as activity triggered by a strong cortical input (1,000–1,500 ms). Single neuron voltage traces are shown for the same simulation (bottom).

We tested SNNbuilder by building two models: a balanced cortical network (Brunel, 2000) showing self-sustained asynchronous-irregular (SSAI) activity (Kriener et al., 2014) and a model of the mouse striatum (Hjorth et al., 2020) reproducing resting state activity (Figure 6).

### 4.1. Self-Sustained Network

Networks of spiking neurons can show SSAI firing under a certain balance of excitatory and inhibitory transmission, with no need for random background input. We reproduced a cortical network model with excitatory and inhibitory populations (Brunel, 2000) and explored the generation and duration of SSAI state based on examples from Kriener et al. (2014).

The model was built following the steps below:

- Login to SNNbuilder (https://snnbuilder.riken.jp) (Figure 7.1).
- Select the target subject, for example, rodent (Figure 7.2).
- Create a model instance using “Build a new model” option (Figure 7.3).
- Specify a model name, scale, and other descriptions (Figure 7.4).
- Select the model name (Figure 7.5) to show tabs for model details (Figure 7.6).
- Select “insert” in the “neurons” tab to load data for neural populations (Figure 7.7).
- Create excitatory and inhibitory neural populations (Figure 7.8).
- Input additional data required in several tabs (Figure 7.9).
- In “Number,” specify *N*_*exc*_ = 10, 000 and *N*_*inh*_ = 2, 500 for excitatory and inhibitory neurons respectively (Figure 8.10).
- Specify post-synaptic potentials (PSPs) for excitatory (AMPA) and inhibitory (GABA) receptors as alpha-functions with a common value of *t*_*V*_*n*__ = 0.5*ms* (rise time of the synaptic function), and PSP amplitudes *V*_*exc*_ = *J* and *V*_*inh*_ = *gJ* (Figure 8.11). This allows exploration of relative inhibitory strength by activating a single pair (i.e., dotted square at Figure 8.11) as a “fixed parameter” while “deactivating” others.
- Set generic values for the neuronal dendritic extent *l*_*x*_ = 600μ*m* (Figure 8.12) and dendrite diameter *d*_*x*_ = 1.6μ*m* (Figure 8.13).
- Implement neural populations as multi-synapse LIF (leaky integrate-and-fire) neurons by the option “import from model” at “Other parameter” tab (Figure 8.14). This enables the selection of a NEST neuron model (Figure 8.15) and transfer of parameters with default values to the neural populations (Figure 8.16).
- Specify connectivity details using the “Projections” tab, with the “insert” function (Figure 9.17).
- Link source and target neural populations and add connectivity specifications by navigating the additional tabs (Figure 9.18).
- Set axonal organization as “diffuse” (Figure 9.19), with a wide spatial domain, in order to emulate random networks in which neurons are independently connected with an equal probability ϵ.
- Define the percentage of source neurons projecting to the target population as 100% (Figure 9.20).
- Assume ϵ = 0.1 to define bouton counts from projections at excitatory neurons as α_{*exc, inh*} → *exc*_ = ϵ × *N*_*exc*_, and at inhibitory neurons as α_{*exc, inh*} → *inh*_ = ϵ × *N*_*inh*_ (Figure 9.21).
- Specify the synaptic location to soma *r*_*x*_ as “proximal” for establishing a minimal PSP attenuation, with a distance within 20% of a generic dendritic extent (Figure 9.22).
- Set a generic redundancy value ρ = 1 (Figure 9.23).
- Implement projections as NEST static synapse models, with default parameter values, similar to the NEST neuron's case (Figure 8.15).
- Create a simulation using the “insert” function (Figure 9.24), specify time resolution *dt* = 0.1*ms*, simulation time for 2, 000*ms*, and spatial organization of neurons in 2D-space with coordinates (*x, y*) randomly generated between [0, 1] and other features (Figure 9.25).
- Add stimuli for the first 1,000 ms as independent Poisson spike trains of constant rate (Figure 9.26).
- Generate model descriptions and simulation code in three sequential steps: get parameters, code building, and files download (Figure 9.27).

**Figure 7:**
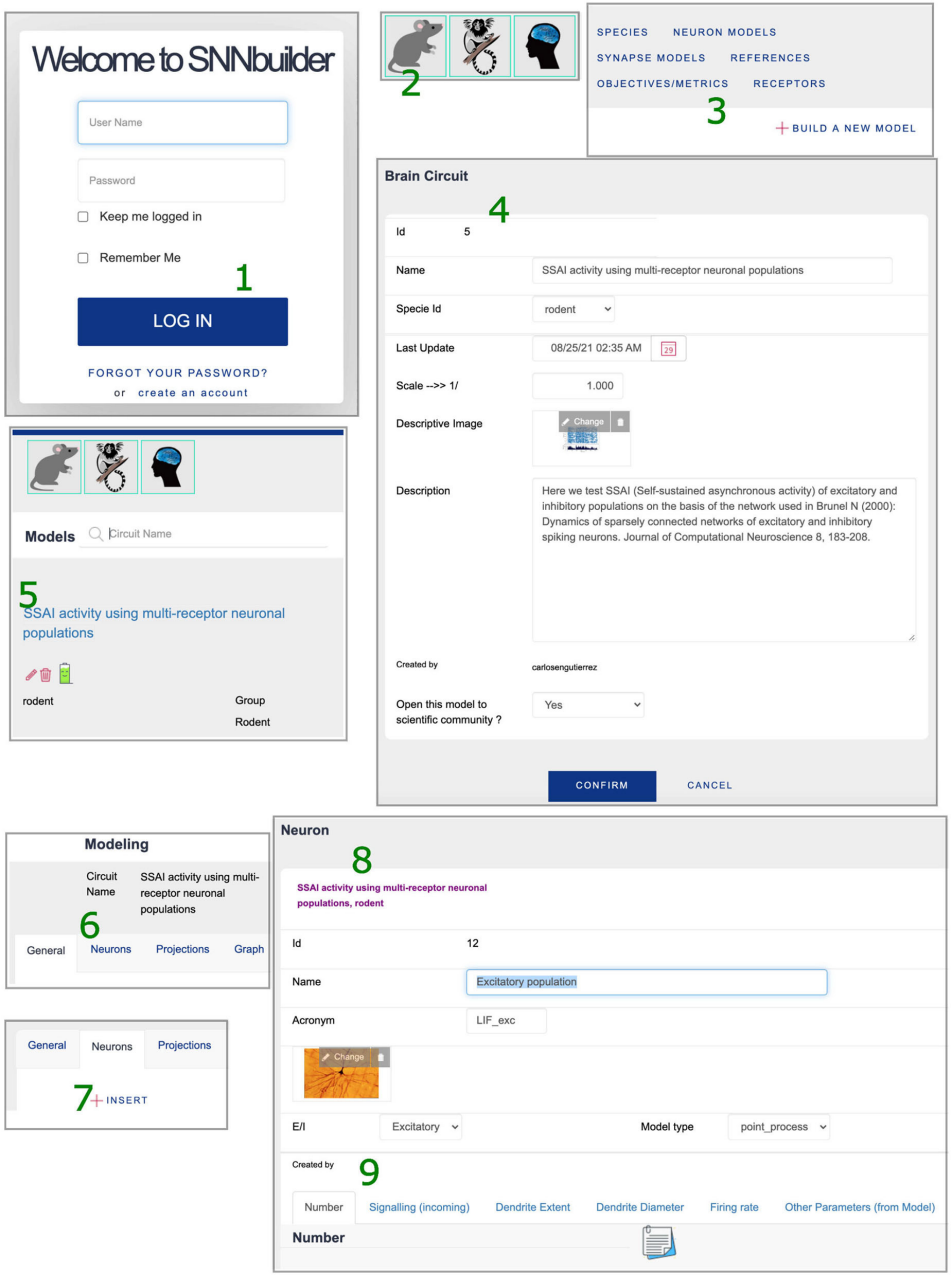
Model creation at SNNbuilder. 1. Login system. 2. Selection of species (subject). 3. “Build a new model” option. 4. Initial specifications. 5. The created model is listed at the front-end. 6. Tabs for modeling workflow. 7. “Insert” option at “Neurons” tab. 8,9. Required specification for neuronal populations.

**Figure 8:**
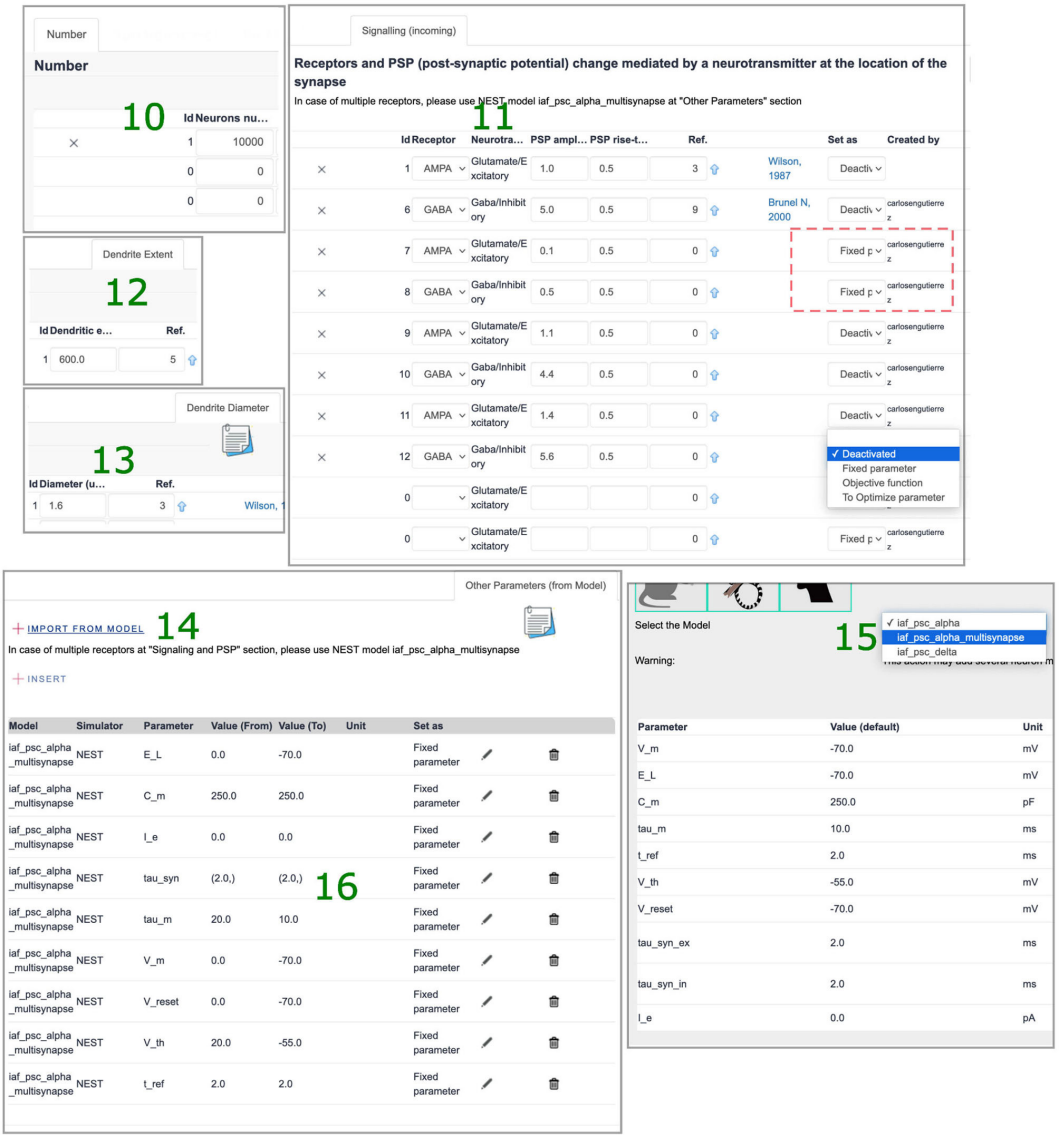
Neuronal population specifications. 10. Number of neurons. 11. Receptors, PSP amplitudes, and rise times. 12,13. Dendrite characteristics. 14. “Import from model” option. 15,16. Selection of a NEST model and migration of default parameters.

**Figure 9:**
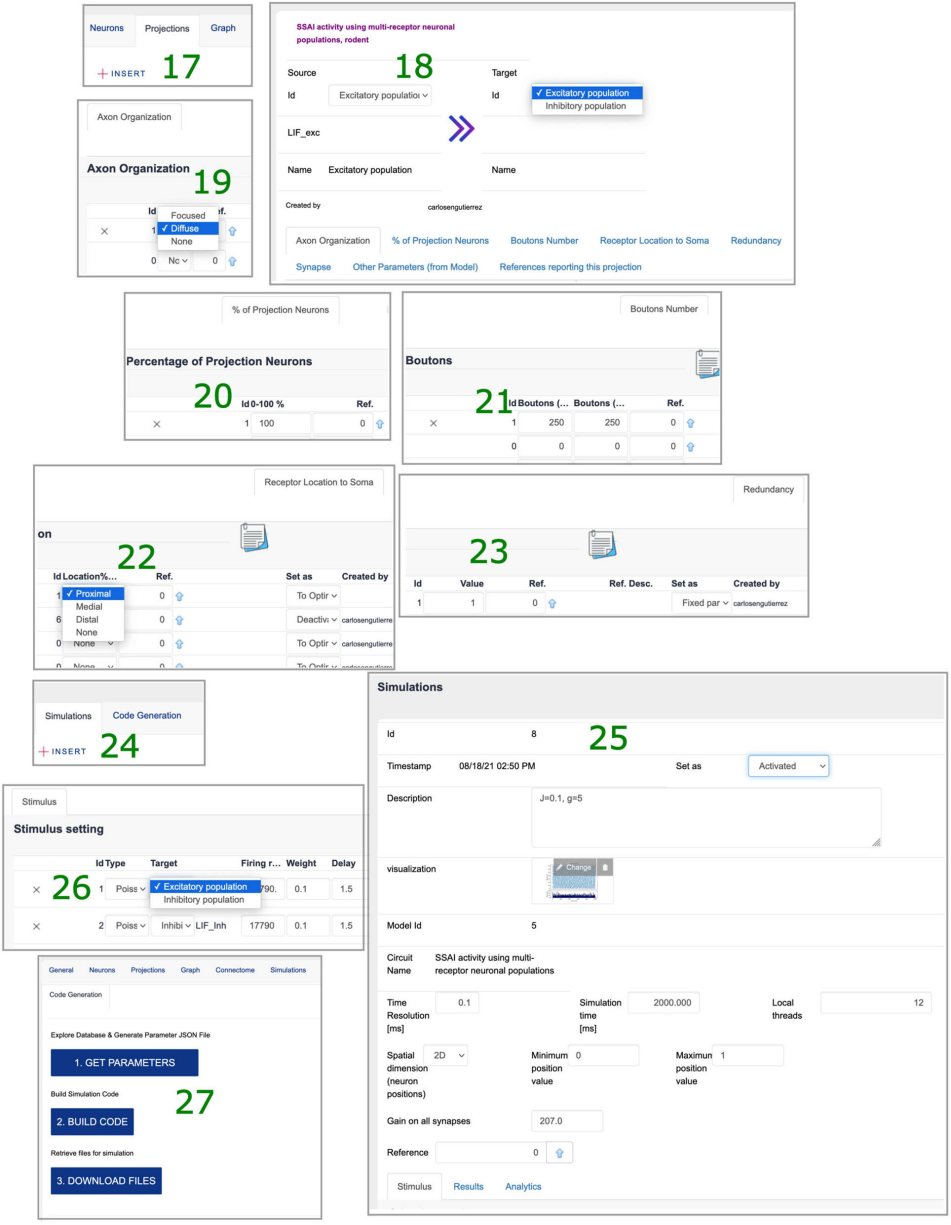
Projection and simulation specifications. 17. “Insert” option at “Projections” tab. 18. Data requirements for network wiring. 19. Axonal organization. 20. Percentage of neurons at source projecting to target. 21. Bouton counts. 22. Synapse location to soma. 23. Redundancy parameter. 24. “Insert” option at “Simulations” tab. 25. Simulation settings. 26. Stimuli to target populations. 27. Model description file and simulation script generation.

Simulations run with different values of *J* and *g* (Figure 6A), for example, *J* = {1.1, 1.4} and *g* = 4 showed different network activities after stimulus offset. While the lifetime for *J* = 1.1 was almost zero, *J* = 1.4 sustained the firing rate, allowing a longer lifetime. Thus, a stronger coupling strength drove the network over the whole simulation time. As reported in Kriener et al. (2014), the SSAI state showed highly irregular spiking activity, with neurons switching between periods of silence or low firing rate, and short bursts or elevated rates, while the average activity of the neural population persisted over the simulation time. It is worth noting that values of *J* and *g* are not directly comparable to those reported by Brunel (2000) and Kriener et al. (2014), since attenuation is applied on the PSP strengths based on dendrite parameters (see Transfer functions in Appendix A).

As an additional test, for the latter parametrization, we observed that the SSAI state is affected by a single change in neuron's morphology: a “distal” location *r*_*x*_ of the receptors in relation to the soma (Figure 9.22) shortened SSAI lifetime (Figure 6A). SNNbuilder easily enabled model changes and code generation for straightforward analyses.

### 4.2. Striatal Microcircuitry

Rodent local striatal microcircuitry has recently been modeled (Hjorth et al., 2020) using the NEURON simulator (Carnevale and Hines, 2006); however, such simulation involves heavy computations due to detailed cell morphologies. We aimed to replicate these results using point-process neurons, which are computationally much less expensive for systematic analysis of model dynamics.

A network was built following data from Hjorth et al. (2020), comprised of 38,237 direct striatal projection neurons (dSPN), 38,237 indirect striatal projection neurons (iSPN), 1,047 fast-spiking (FS) interneurons, 644 low-threshold spiking (LTS) interneurons, and 886 cholinergic interneurons (ChIN). All neuronal types were implemented as LIF with AMPA and GABA receptors, with PSPs modeled as alpha-functions with specific amplitude values for each connection. PSPs were specified as connection weights at the projection synapse parameters, rather than at the neuron receptor level. The axonal delay was assumed generic for all the connections, equal to 0.2*ms*. Neuron parameters, such as membrane time constant, threshold, and resting membrane voltage were taken from Johansson and Silberberg (2020).

Neurons were uniformly distributed in a 1*mm*^3^ volume, matching the neuronal density in the striatum (Rosen and Williams, 2001). Connections were created using a fixed in-degree rule and a spherical mask with size based on axonal and dendritic field diameters. Other connection-related parameters, such as bouton number, the distance between soma and synapse, and the number of synapses from a single source were also taken and calculated by Hjorth et al. (2020).

Two levels of external input were modeled in the network: the first 1,000 ms of simulation correspond to 2 Hz glutamatergic baseline activity from the cortex and thalamus. In order to simulate synaptic input, all neurons were assumed to have 150 AMPA synapses, all receiving independent inputs that can be modeled as a 300 Hz Poisson spike train, similar to what is described in Hjorth et al. (2009). The next 500 ms correspond to higher-level cortical activity, defined as an 8*Hz* glutamatergic input, and modeled as a 1,200 Hz Poisson spike train superimposed on the baseline input train, after which baseline activity is restored.

The workflow final step, the code generation, provided a python script for simulations. An optimization step, not implemented by the current SNNbuilder release, was performed for this modeling example (see Current limitations). Both local (inhibitory) and external input (excitatory) weights to each population were optimized simultaneously, using grid search and a custom multi-objective function: for each population and for each stimulation regime, a target range of plausible firing rates was defined, and error was defined as the normalized distance to the center of that interval. The set of weights that minimized this error was then selected, with the firing rates of all populations matching those described in Hjorth et al. (2020).

The optimization process, first, found weights that yielded good behavior for the baseline activity (from 0 to 1,000 ms, Figure 6B). Once optimized, these values were active during the whole simulation (from 0 to 1,500 ms). Then, new Poisson generators were introduced corresponding to a higher level of cortical activity (from 1,000 to 1,500 ms) and whose weights were optimized while keeping the baseline ones fixed.

A raster plot of the network activity after optimization is shown in Figure 6B and voltage traces (*mV*) of single neurons, in which the two levels of activity are distinguishable.

## 5. Discussion

Modeling the brain is a challenge that requires collective effort. Large-scale cohesion of researcher knowledge, ideas, publications, and experimental data can be realized on the internet, where humans are hyper-connected, constituting a convenient frame for brain modeling. We have designed SNNbuilder as a web-application to support the collaborative building of sustainable, renewable, and scaleable SNN models.

The introduced framework organizes specifications to model any region of the brain through a straightforward GUI (Figure 3). Anatomical, morphological, and physiological data are systematically loaded and updated, and their passage as neural and synaptic parameters is managed by transfer functions (see Appendix A). A generic relational database (Figure 2A) is designed to accommodate accumulating data and includes references and notes to accurately acknowledge data sources and to trace model details (Supplementary Figure B.2). SNNbuilder workflow (Figures 3, 7, 8, 9) was tested on two model examples: a self-sustained asynchronous irregular network and a model of mouse striatal circuitry (Figure 6).

Major data sources are scientific publications. Paper surveys require the identification of relevant parameters for modeling, which is time-consuming for humans. Efforts are ongoing to extract data from a large collection of literature and to store the data in open databases. For example, Bjerke et al. (2020) standardized and quantified information about cellular parameters in the murine basal ganglia from public repositories, and Tripathy et al. (2014) extracted electrophysiological properties of diverse neuron types from existing literature. The desired future extension is SNNbuilder compatibility with open database sources, not only for consuming plain data but also for incorporating automatic discovery of parameters by text-mining algorithms and knowledge-graph building.

Compatibility with resources, such as EBRAINS, Brain/MINDS, and NeuroML, are crucial for improving the modeling process. We aim for SNNbuilder-to-application and SNNbuilder-to-databases compatibility, so system input(s) and output(s) can be shared and integrated. A preliminary effort corresponds to the SNNbuilder capability to import NEST models and connectomic data from JSON files, including remotely located files for the latter case (Figure 4 and Supplementary Figure B.1). By this functionality, upon the opening of data, marmoset connectomic retrieval by a web service at Brain/MINDS is possible in the short term. This will provide full or partial data for loading, automatically, neurons and projections in SNNbuilder. An architecture composed of web-services or APIs for straightforward access to SNNbuilder data and models, and web-services for data consumption from open sources (Supplementary Figure B.1) is required and considered as future challenge. Moreover, an important standard supporting data sharing across brain projects is Neurodata Without Borders (NWB, Teeters et al., 2015; Rübel et al., 2021). SNNbuilder management of inputs/outputs in NWB format will be considered as well in future releases.

New system functions and features are required as well and included in future work, especially needed for building complex experimental settings and detailed models. For example, parameter definition based on distributions, stimuli protocols associated with objectives or metrics, integration of functional-related metadata, re-use of parameters from other models, new transfer functions and more connection rules, model versioning and history tracking, online simulation management, and parameter optimization.

Our generic database structure supports the future implementation of an optimization engine. Optimization will preserve parameters labeled as “fixed” while exploring “to-optimize” parameters within defined value intervals, assessing model activity against data labeled as “objectives.” The generic character of data-to-model conversion will allow comparisons across species as well, since models for different subjects along with their simulation results can be compared on the same dimensions.

A further challenge is code generation for multiple simulators, which may require the development of new mapping processes (transfer functions). On this, compatibility with NeuroML, a simulator-free approach for model description may provide strong advantages. Currently, the SNNbuilder model description in JSON format corresponds to dictionaries listing the specifications. That output could be prepared in compatible XML (Extensible Markup Language) format, following the standard NeuroML. Model description in NeuroML enables simulations on different tools, avoiding code generation/preparation for multiple simulators. Having that advantage, model specifications can be prepared to target biophysical neuron models and complex networks; thus, SNNbuilder may support the state-of-the-art NEURON simulator. Our database will be extended in such a case, for the inclusion of new data entities like detailed morphology and ion channels.

SNNbuilder plans to enable online simulation and result analytics, by simulating models on the server and visualizing results *via* the web-browser, as shown in Spreizer et al. (2021). That will facilitate immediate building-testing iterations for an intuitive understanding of model dynamics. Big spike data could be loaded into the database and meticulously queried and plotted for better interpretation of results. While small simulations could be triggered instantly, large simulations can be prepared and dispatched for high-performance computing, as shown in Feldotto et al. (2022). SNNbuilder straightforward code generation may include job scripts for the setup and simulation of large-scale models on the Fugaku supercomputer (Sato et al., 2020), as well on collaborative simulation infrastructures like Fenix from EBRAINS (Alam et al., 2019). Compatibility with distributed computational resources facilitates the access and usage of services and is included in our future challenges.

As an introductory video from the International Brain Initiative observed, “It takes the world to understand the brain. It is the most complex organ in the human body” (Adams et al., 2020). Understanding the brain requires not only biological data but also tools to enable the engagement of a diversity of researchers, with different backgrounds and opinions, to support independent, free contribution of ideas. Our framework supports that collaboration for modeling the brain.

## Data Availability Statement

SNNbuilder is currently running on the internet and is available at: https://snnbuilder.riken.jp. A test user for tool exploration and code generation of existing models is available (user: testuser password: snnbuilder). For full privilege users, please send a request to carlos.gutierrez@oist.jp. Source code and data base structure are available upon request. The authors of this work are open to discussion and collaboration. Feedback for improving our work is gladly welcome and appreciated.

## Author Contributions

CG designed and developed SNNbuilder, modeled a self-sustained network example, and ran simulations. HS helped with the framework compatibility with Brain/MINDS connectomic data, and GUI design. HM modeled the striatal circuitry example, ran simulations and optimizations, and supported debugging. KD helped with SNNbuilder concept, design, and future integration within the International Brain Initiative (IBI). All authors contributed to the writing of the manuscript.

## Funding

This research was supported by the Collaboration Research for Development of Techniques in Brain Science Database Field and the Collaborative Technical Development in Data-driven Brain Science grants from RIKEN Center for Brain Science, the program for Brain Mapping by Integrated Neurotechnologies for Disease Studies (Brain/MINDS) JP18dm0207030 and 21dm0207001 from the Japan Agency for Medical Research and Development (AMED), the Post-K Application Development for Exploratory Challenges (hp160266, hp170251, hp180223, and hp190157) from Ministry of Education, Culture, Sports, Science and Technology of Japan (MEXT), the KAKENHI Grant 16H06563 from Japan Society for the Promotion of Science (JSPS), and internal funding from the Okinawa Institute of Science and Technology Graduate University to KD.

## Conflict of Interest

The authors declare that the research was conducted in the absence of any commercial or financial relationships that could be construed as a potential conflict of interest.

## Publisher's Note

All claims expressed in this article are solely those of the authors and do not necessarily represent those of their affiliated organizations, or those of the publisher, the editors and the reviewers. Any product that may be evaluated in this article, or claim that may be made by its manufacturer, is not guaranteed or endorsed by the publisher.
