# Supplementary material for: A Spiking Neural Network Builder for Systematic Data-to-Model Workflow
Source: Front Neuroinform. 2022 Jul 13;16:855765. doi: 10.3389/fninf.2022.855765 (PMC9326306; doi:10.3389/fninf.2022.855765)
Supplement: Supplementary file 1 [file Presentation_1.pdf]

frontiers

Supplementary Material

APPENDIX A TRANSFER FUNCTIONS

While some data values are mapped directly as NEST parameters, others are processed by transfer functions. The transformations are based on previous work from Deco et al. (2008), Deco et al. (2008); Liénard and Girard (2014); Girard et al. (2020)). The following transfer functions were included:

1. Neuron in-degree:
   The number of neurons from a source population providing inputs to a single neuron in a target population is estimated as the number of input synapses $\nu_{x \leftarrow y}$, also known as the in-degree of the target neuron:
$$\nu_{x \leftarrow y} = \frac{P_{Y \to X} N_Y}{N_X} \cdot \alpha_{y \to x} \tag{S1}$$
   Where Y and X are the source and target populations respectively, N_Y and N_X the corresponding number of neurons, $P_{Y \to X}$ the proportion of neurons in Y projecting to X, $\alpha_{y \to x}$ the average number of synapses each neuron of Y makes in X. The in-degree is used for connection rules of the type fixed in-degree (Hahne et al. (2021)).
2. Neuron out-degree:
   $\alpha_{y \to x}$ is approximated by the synaptic bouton counts, frequently found on single-axon tracing studies. Besides being used to define the in-degree value described above, $\alpha_{y \to x}$ is also used stand-alone for connection rules of the type fixed out-degree.
3. Redundancy:
   Considering that a source-target neuron pair may have multiple synaptic contacts, an average redundancy $\rho_{Y \to X}$ is considered (Girard et al. (2020)). Redundancy is a number between 1 (each synapse comes from a different neuron) and $\nu_{x \leftarrow y}$ (a single neuron provides all synapses), and it is used to adjust the in-degree: $\nu_{x \leftarrow y} / \rho_{Y \to X}$.
   Connection rules of the type "in-degree" draw $\nu_{x \leftarrow y} / \rho_{Y \to X}$ neurons of Y, within a pre-defined spatial mask (i.e. a circular mask), and connect them to each neuron of X. There is a trade-off between the neural population size (number of neurons), the size of the spatial mask and the redundancy value. The connection mask should allocate enough neurons in order to achieve the required in-degree of the connection rule, if not, a higher redundancy adjusts the in-degree accordingly to the available number of neurons within the mask. Similarly, this can be applied to the out-degree-based connectivity.
   Adjustment by redundancy is automatic if $\rho_{Y \to X}$ is specified (Fig 9.23). By default, redundancy is 1 and no adjustment occurs. Testing several values of the redundancy is possible; however, it is required to change or activate/deactivate values by GUI, and re-generate the simulation code.
   Redundancy is also useful to lighten computations when scaling to larger models, allowing, relatively, conservation of model's properties (see Connection weight below); although limitations on the neural dynamics may occur (Van Albada et al. (2015)). Higher redundancy creates fewer synapses, and adjusts the synaptic weight to maintain a similar network activity.
4. Dendritic attenuation:
   Dendritic attenuation $\gamma_{x \leftarrow y}$ is considered as a function of the mean diameter d_x and the average maximal extent l_x of the dendrite, as well as the mean distance r_x where synaptic contacts are made to the soma, expressed as percentage of l_x. Attenuation is computed as:

$$\gamma_{x \leftarrow y} = \frac{\cosh\left(L_x(1 - r_x)\right)}{\cosh L_x} \;,\;\; with\; L_x = l_x \sqrt{\frac{4}{d_x}\frac{R_i}{R_m}} \tag{S2}$$

With L_x as the dendrite-based electronic constant (Koch (2004)), and R_i & R_m the intracellular and membrane resistances respectively.

5. Connection weight:
   The incoming post-synaptic potential change caused by a single spike at the location of the synapse V_n (mV) is attenuated by $\gamma_{x \leftarrow y}$ on its way to the soma, and adjusted by the redundancy $\rho_{Y \rightarrow X}$. The connection weight w_n is then computed as:

$$w_n = \rho_{Y \rightarrow X} V_n \gamma_{x \leftarrow y} \tag{S3}$$

   Beside PSP-based connection weights, an alternative approach for defining connection weights is available at the synapse-level parameters (synapse tab) or within NEST synapse model (other parameters tab, Fig.9.18). The connection weight is not attenuated when ”None” is assigned to the parameter ”Location to Soma” at the “Receptor & Location to soma” tab (Fig.9.18) in Projections.
6. PSP rise time: depending on the receptor type, the PSP rise time t_{V_n} is used as the tau_{syn} parameter of the synaptic alpha function included in several NEST neuron models.
7. Connection parameters: connection rules of the type ”in-degree” use $\nu_{x \leftarrow y}$ as the number of incoming connections to a single target neuron. Whereas, rules of the type ”out-degree” adopt $\alpha_{y \rightarrow x}$ as the number of outgoing connections from a single source neuron. Rules of the type ”constant probability” and ”distance-depended Gaussian probability”, use p (probability) and std (standard deviation) respectively, which are parameters specified by GUI. SNNbuilder implements topological connection rules for NEST 3. The spatial mask, which defines the subset of neurons considered as potential targets (or sources) for each source (target) neuron, is assumed as the axonal spatial domain (focused or diffuse), circular or spherical (2D or 3D spatial organization), of configurable radius. Other parameters such as connection strength w_n and axonal delay, are mapped to connection rules as well.

APPENDIX B SUPPLEMENTARY FIGURES

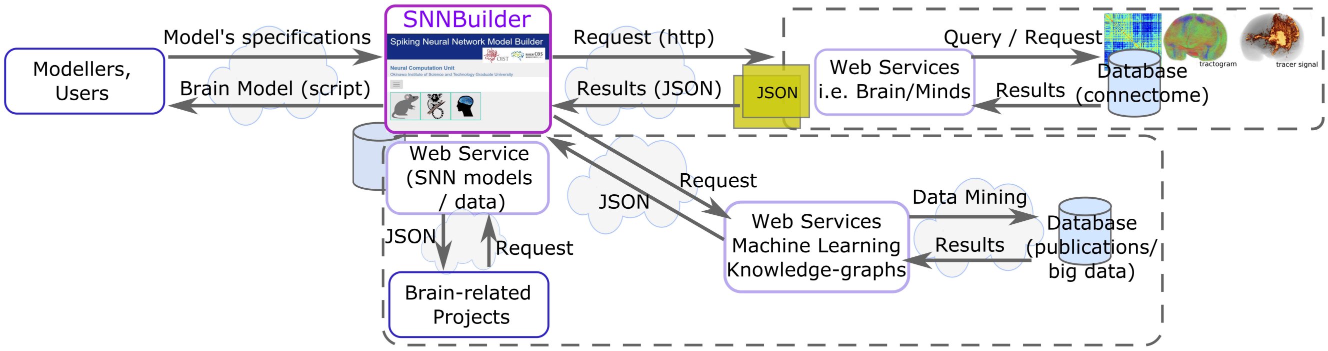

Figure B.1. SNNbuilder incorporates a function to import connectomic data (in JSON format) from remote URL, avoiding the manual specification of neurons and projections. Non-enclosed area corresponds to the current SNNbuilder release. Future work (dotted squares) targets application-to-application integration in real time and cross-collaboration; for example, connectomic data retrieval by a web-service at Brain/MINDS, data exchange as provider/consumer of web-services to/from other projects, machine learning services, knowledge-graphs, and others.

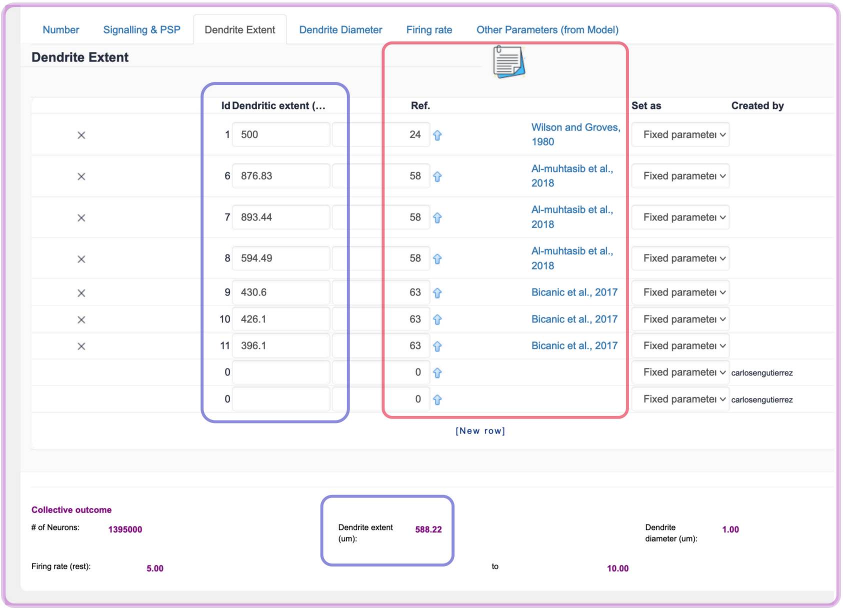

Figure B.2. Several values surveyed for the dendritic extent of a neuron type serve as the ”collective” knowledge, computed as the mean value (blue squares). References are recorded as well, linking the original sources by DOI’s (red square). Notes, memos or reminders support model documentation (red square, top side).
